# Impact of non-neurological complications in severe traumatic brain injury outcome

**DOI:** 10.1186/cc11243

**Published:** 2012-03-12

**Authors:** Luisa Corral, Casimiro F Javierre, Josep L Ventura, Pilar Marcos, José I Herrero, Rafael Mañez

**Affiliations:** 1 Intensive Care Unit, Hospital Universitari de Bellvitge, Feixa Llarga s/n, L'Hospitalet de Llobregat-08907, Barcelona, Spain; 2 Departament de Ciències Fisiològiques II, Universitat de Barcelona, Feixa Llarga s/n, L'Hopitalet de Llobregat-08907, Barcelona, Spain; 3 Intensive Care Unit, Hospital Universitari Germans Trias i Pujol, Carretera de Canyet s/n, Badalona-08916, Barcelona, Spain; 4 Institut d'Investigació Biomèdica de Bellvitge (IDIBELL), Feixa Llarga s/n, L'Hopitalet de Llobregat-08907, Barcelona, Spain

## Abstract

**Introduction:**

Non-neurological complications in patients with severe traumatic brain injury (TBI) are frequent, worsening the prognosis, but the pathophysiology of systemic complications after TBI is unclear. The purpose of this study was to analyze non-neurological complications in patients with severe TBI admitted to the ICU, the impact of these complications on mortality, and their possible correlation with TBI severity.

**Methods:**

An observational retrospective cohort study was conducted in one multidisciplinary ICU of a university hospital (35 beds); 224 consecutive adult patients with severe TBI (initial Glasgow Coma Scale (GCS) < 9) admitted to the ICU were included. Neurological and non-neurological variables were recorded.

**Results:**

Sepsis occurred in 75% of patients, respiratory infections in 68%, hypotension in 44%, severe respiratory failure (arterial oxygen pressure/oxygen inspired fraction ratio (PaO_2_/FiO_2_) < 200) in 41% and acute kidney injury (AKI) in 8%. The multivariate analysis showed that Glasgow Outcome Score (GOS) at one year was independently associated with age, initial GCS 3 to 5, worst Traumatic Coma Data Bank (TCDB) first computed tomography (CT) scan and the presence of intracranial hypertension but not AKI. Hospital mortality was independently associated with initial GSC 3 to 5, worst TCDB first CT scan, the presence of intracranial hypertension and AKI. The presence of AKI regardless of GCS multiplied risk of death 6.17 times (95% confidence interval (CI): 1.37 to 27.78) (*P *< 0.02), while ICU hypotension increased the risk of death in patients with initial scores of 3 to5 on the GCS 4.28 times (95% CI: 1.22 to15.07) (*P *< 0.05).

**Conclusions:**

Low initial GCS, worst first CT scan, intracranial hypertension and AKI determined hospital mortality in severe TBI patients. Besides the direct effect of low GCS on mortality, this neurological condition also is associated with ICU hypotension which increases hospital mortality among patients with severe TBI. These findings add to previous studies that showed that non-neurological complications increase the length of stay and morbidity in the ICU but do not increase mortality, with the exception of AKI and hypotension in low GCS (3 to 5).

## Introduction

Severe Traumatic Brain Injury (TBI) mortality and morbidity is frequently caused by the neurological consequences of the brain injury [1,2]. Nevertheless, non-neurological complications are also frequent, and may be cardiovascular, respiratory, infectious and others [3-5]. An initial study by Zygun *et al. *showed that non-neurological organ dysfunction measured by Sequential Organ Failure Assessment (SOFA) was not associated with increased mortality in neurocritical patients with TBI or subarachnoid hemorrhage, despite the frequent finding of cardiopulmonary dysfunction [6]. However, a later study by the same group associated non-neurological organ dysfunction with worse outcome, reporting that cardiovascular and respiratory complications were the most common dysfunctions [5].

The pathophysiology of systemic complications after TBI is unclear. Complications may arise from the direct effect of the injury or as a result of the side effects of therapy [7,8]. Neurogenic causes such as the massive catecholamine and neuro-inflammatory response associated with brain injury may contribute to systemic complications. In addition, the intensive care management of TBI patients is mainly directed at neurological problems and may contribute to non-neurological complications [8].

A better knowledge of the incidence, causes and consequences of non-neurological complications in patients with severe TBI would help in their prevention, treatment and prognosis. The purpose of this study was to analyze the non-neurological complications presented by patients with severe TBI admitted to the ICU, the impact of these complications on hospital mortality and outcome at one year, and their possible correlation with TBI severity.

## Materials and methods

The study included 224 adult patients with severe TBI (initial Glasgow Coma Scale (GCS) < 9), consecutively admitted to the ICU, between 1998 and 2004. It is an observational retrospective cohort study. The initial GCS score was obtained upon admission to the emergency ICU. If the patient was already intubated and on mechanical ventilation the previous GCS score was used and GCS scale was divided into two levels of severity: low (GCS 3 to 5) and high (GCS 6 to 8) [9,10]. The study was approved by the institution's Research Ethics Committee, which did not consider informed consent to be necessary because of the study design. Management of patients followed international guidelines [11], with the goal of maintaining a cerebral perfusion pressure (CPP) of 60 to 70 mmHg and an intracranial pressure (ICP) of < 20 mmHg. Intracranial hypertension was defined as ICP > 20 mmHg for 15 minutes without a systemic cause. Elevations in ICP were managed sequentially with sedation, paralysis, mannitol and mild hyperventilation. Barbiturate therapy was considered for refractory intracranial hypertension. The therapy index [12] was used to quantify the treatment for intracranial hypertension that was enough to quantify the treatments used in that year following international guidelines. Computerized tomography (CT) of the brain at admission was evaluated following the Traumatic Coma Data Bank Computed Tomography (TCDB CT) scan classification. Mortality at hospital discharge and Glasgow Outcome Scale (GOS) at one year was obtained and was dichotomized as worse outcome (dead, vegetative state and severe disability) and as good outcome (moderate disability and good recovery) [9].

The following non-neurological complications were recorded:

Cardiovascular: ICU hypotension (systolic blood pressure (SBP) < 90 mmHg for 30 minutes during the ICU stay), hypertension (SBP > 160 mmHg for more than 30 minutes and, in hypertensive subjects, an increase > 40 mmHg that required treatment change [3]), cardiac arrhythmias (bradycardia < 60 beats/minute or tachycardia > 120 beats/minute) and the need for vasoactive drugs (dopamine, dobutamine or norepinephrine).

Respiratory: infectious respiratory complications (aspiration, tracheobronchitis or pneumonia (infiltrate on chest radiograph and analysis of gram or sputum culture-positive [3])), acute respiratory distress syndrome (ARDS) [13], PaO_2_/FiO_2 _ratio 200 to 300 and severe respiratory failure (SRF) as PaO_2_/FiO_2 _ratio < 200.

Septic: severe sepsis [14] and septic shock [14].

Renal: acute kidney injury (AKI) (serum creatinine > 150 μmol/L) with or without dialysis [3].

Abdominal/digestive complications: ileus, cholecystitis, bilirubin levels > 18 μmol/L and aspartate amino transferase (AST) > 1 μkat/L [3].

Endocrinometabolic: hyponatremia < 130 mmol/L, hypernatremia > 150 mmol/L, syndrome of inappropriate antidiuretic hormone secretion (SIADH) (Na < 130 mmol/L, urine osmolality exceeding serum osmolality [3]), cerebral salt wasting syndrome (excessive diuresis with urine sodium below > 20 mmol/L [15]) and diabetes insipidus (diuresis > 200 mL/hour for 24 hours without response to fluid restriction or needing treatment with desmopressin, urine specific gravity < 1,005 and a urine osmolality less than half that of plasma).

Bleeding: hemorrhage needing blood transfusion products (> 4 packed cells) and/or hemorrhagic shock during ICU admission.

### Statistical analysis

Continuous variables (age, ICU stay and in hospital stay) were expressed as median and interquartile range. Categorical data were expressed as frequency and percentage. We used the chi square test to compare categorical data and proportions, and the T-test, Mann-Whitney test or Kruskal-Wallis, as appropriate, to compare continuous variables. Multivariate analysis was performed with multivariate regression and variables were selected using either the stepwise or the backward regression procedures. Confounding and interaction variables were studied, and the effect size on outcome was calculated for clinical complications during the ICU stay, as well as the effect size on ICU hypotension. An alpha level of 0.05 was used to determine statistical significance. All data in the present study were analyzed using SPSS version 16.0 (SPSS Inc, Chicago, USA).

## Results

The characteristics of the sample are shown in Table 1[9,16]. ICP was monitored in 73% of the patients (*n *= 164), 51% (*n *= 83) of whom developed intracranial hypertension and 56% low cerebral perfusion pressure at some point in the evolution of their condition. Hypoxia at admission was detected in 64 patients, it was not detected in 152 and 8 had missing data. Hypotension at admission was detected in 38 patients. The median therapy index score was 3, with 50% of the patients ranging between 2 and 7. The median ICU stay was 15 days (interquartile range 20).

**Table 1:** Clinical characteristics of 224 patients with severe traumatic brain injury.

		**No. (%)**
		
		224
Age (median, interquartile range)		35.6 (23-55)
Gender	Male	189 (84)
	Female	35 (16)
Mechanism of injury	Traffic accident	148 (66)
	Falls	58 (26)
	Assault	6 (3)
	Others	12 (5)
Multiple trauma		127 (57)
GCS	3	30 (13)
	4	35 (16)
	5	30 (13)
	6	40 (18)
	7	35 (16)
	8	54 (24)
	Low GCS (GCS 3, 4, 5)	95 (42)
	High GCS (GCS 6, 7, 8)	129 (58)
TCDB CT scan classification	I	14 (6)
	II	120 (54)
	III	26 (12)
	IV	21 (9)
	EM	38 (17)
	NEM	5 (2)
	I, II, EM	172 (77)
	III, IV, NEM	52 (23)
Detected hypotension at admission		38 (17)
Detected hypoxia at admission		64 (29)
Detected intracranial hypertension		83 (37)
Pupil abnormalities		76 (34)
Dead	At ICU discharge	67 (31)
	At hospital discharge	74 (33)
	At 6 and 12 months	79 (35)
Days in ICU (median, interquartile range)		15 (7-26)
Days in hospital (median, interquartile range)		26 (11-49)

EM: evacuable mass, GCS: Glasgow Coma Scale, ICU: Intensive Care Unit, NEM: non-evacuable mass, TCDB CT: Traumatic Coma Data Bank Computed tomography.

TCDB CT was grouped in two groups: one best TCDB CT scan classification type I, II and evacuable mass (EM) and the other worst TCDB CT scan type III, IV and non-evacuable mass (NEM).

Non-neurological complications throughout the ICU stay are shown in Table 2: sepsis occurred in 75% of the patients, respiratory infections in 68%, ICU hypotension in 44%, severe respiratory failure (PaO_2_/FiO_2 _< 200) in 41% and AKI in 8%. Vasoactive drugs were used in 96% of the patients with ICU hypotension. There was only one patient with previous chronic renal failure, which was not included as AKI. The univariate analysis of the non-neurological complications showed that hypotension, severe respiratory failure, septic shock, AKI, bleeding complications and non-neurological surgery were prognostic factors related to hospital mortality (Table 3). A multivariate regression analysis was performed for hospital mortality and GOS at one year post-TBI, taking into account variables with clinical relevance (age), variables related to TBI severity [(initial GCS (divided into GCS 3 to 5 and GCS 6 to 8), first CT scan (TCDB) and the presence of intracranial hypertension) and non-neurological complications that were statistically significant in the univariate analysis. The multivariate analysis showed that the worst outcome at one year was independently associated with age, initial GCS 3 to 5, worst TCDB first CT scan and the presence of intracranial hypertension but not AKI (Table 4). The multivariate analysis showed that hospital mortality was independently associated with an initial GSC 3 to 5, a worst TCDB first CT scan, the presence of intracranial hypertension and AKI (Table 5). Neither multiple trauma nor chest trauma was independently associated with higher mortality or worse outcome. Seventeen patients (8%) developed AKI and 13 of them (76%) died. In 10 of the 13 patients (77%) death occurred after day 7 of ICU admission and in 4 of 13 renal replacement techniques had been used.

**Table 2:** Non-neurological complications.

Variables		n (%)
Respiratory	Respiratory infections	152 (68)
	Atelectasis	47 (21)
	ARDS	20 (9)
	PaO_2_/FiO_2 _< 200	92 (41)
	PaO_2_/FiO_2 _200-300	94 (42)
Cardiovascular	Hypotension	99 (44)
	Hypertension	28 (12)
	Arrhythmias	25 (11)
	Dopamine, dobutamine or norepinephrine	156 (70)
Infection	Sepsis	169 (75)
	Septic shock	13 (6)
AKI		17 (8)
Abdominal complications		40 (18)
Electrolytical complications		48 (21)
Bleeding complications		60 (27)
Non-neurosurgical surgery (traumatic, digestive, maxillar or plastic)		62 (28)

AKI: acute kidney injury, ARDS: acute respiratory distress syndrome, PaO_2_/FiO_2_: arterial oxygen pressure/oxygen inspired fraction ratio.

Respiratory infections: aspiration, tracheobronchitis and pneumonia were grouped,. Non-neurosurgical surgery: traumatic, digestive, maxillar or plastic surgery.

**Table 3:** Non-neurological complications associated to hospital mortality.

Variables		Deaths 74 (33%) n (%)	Survive 150 (67%) n (%)	*P*
Respiratory	Respiratory infection	45 (30)	107 (70)	NS
	Atelectasis	11(23)	36 (77)	NS
	ARDS	18 (90)	2 (10)	< 0,01
	PaO_2_/FiO_2 _< 200	43 (47)	49 (53)	< 0,05
	PaO_2_/FiO_2 _200-300	21 (22)	73 (78)	< 0,05
Cardiovascular	Hypotension	50 (51)	49 (49)	< 0,05
	Hypertension	7 (25)	21 (75)	NS
	Arrhythmias	12 (48)	13 (52)	NS
	Dopamine, dobutamine or norepinephrine	63 (40)	93 (60)	< 0,05
Infection	Sepsis	53 (31)	116 (69)	NS
	Septic shock	11 (85)	2 (15)	< 0,05
AKI		13 (76)	4 (24)	< 0,05
Abdominal complications		32 (30)	76 (70)	NS
Electrolytical complications		18 (38)	29 (62)	NS
Bleeding complications		30 (50)	30 (50)	< 0,05
Non-neurosurgical surgery		13 (21)	48 (79)	< 0,05

AKI: acute kidney injury, ARDS: acute respiratory distress syndrome, PaO_2_/FiO_2_: arterial oxygen pressure/oxygen inspired fraction ratio. Respiratory infections: aspiration, tracheobronchitis and pneumonia were grouped.

**Table 4:** Logistic regression backward stepwise for worse outcome at one year.

Variable	OR	IC 95%	*P *value
Age	1.03	1.01-1.06	0.007
GCS 3-5	3.26	1.36-7.89	0.008
Worst TCDB first CT scan	3.05	1.15-8.12	0.025
Intracranial hypertension	3.83	1.72-8.53	0.001

GCS: Glasgow Coma Scale, IC 95%: 95% confidence interval for the OR, OR: odds ratio, TCDB CT: traumatic coma data bank computed tomography.

Worst TCDB CT scan referring to type III, IV and non-evacuable mass.

**Table 5:** Logistic regression backward stepwise for mortality.

Variable	OR	IC 95%	*P *value+
GCS 3-5	2.47	1.03-5.93	0.043
Worst TCDB first CT scan	3.69	1.43-9.51	0.007
Intracranial hypertension	10.18	3.85-26.89	0.000
Renal failure	7.12	1.58-31.98	0.010

GCS: Glasgow Coma Scale, IC 95%: 95% confidence interval for the OR, OR: odds ratio, TCDB CT: traumatic coma data bank computed tomography.

Worst TCDB CT scan referring to type III, IV and non-evacuable mass.

The specific effect of ICU hypotension, severe respiratory failure or AKI on outcome was also assessed in a multivariate regression model that included age, initial GCS, initial CT scan (TCDB), the presence of intracranial hypertension, the two other non-neurological complication(sepsis and respiratory infections) and their interactions. The presence of AKI regardless of GCS multiplied the risk of death 6.17 times with 95% CI of 1.37 to 27.78 (*P *< 0.02) and ICU hypotension in patients with low initial GCS (3 to 5) significantly increased the risk of death (4.28 times with 95% CI of 1.37 to 27.78, *P *< 0.05) (Table 6).

**Table 6:** Adjusted odds ratio for predictors of mortality and adjusted odds ratio for predictors of ICU hypotension in our cohort.

	Variable	OR	IC 95%	*P *value
Mortality	ICU hypotension with low GCS 3-5	4.28	1.22-15.07	0.024
	ICU hypotension with high GCS 6-8	0.13	0.01-02.33	0.167
	Severe respiratory failure	1.39	0.57-03.35	0.467
	Renal failure	6.17	1.37-27.78	0.018
ICU hypotension	GCS 3-5	3.37	1.66-6.82	0.001

GCS: Glasgow Coma Scale, IC 95%: 95% confidence interval for the OR, ICU: intensive care unit, OR: odds ratio.

To estimate the effect of initial GCS on the development of non-neurological complications a multivariate regression analyzed the interactions between these conditions, finding a single interaction, low initial GCS (3 to 5), multiplied the risk of developing ICU hypotension 3.37 times (*P *< 0.005) (Table 6), suggesting that the latter condition was related to the neurological situation.

Finally, we also observed the impact of non-neurological complications in the ICU stay of surviving patients with severe TBI. The ICU stay (median (interquartile range)) in surviving patients was longer when hypotension (16 (17) versus 30 (27) days, *P *< 0.01), severe respiratory failure (16(20) versus 25 (28) days, *P *< 0.01) and AKI (18 (20) versus 39 (39) days, *P *< 0.05) were present.

## Discussion

The present study found that non-neurological complications were frequent during the ICU stay in a cohort of patients with severe TBI. We found some differences vis-à-vis other studies with regard to non-neurological conditions leading to worse prognosis. Hypotension, pneumonia, infectious complications and coagulation dysfunction have been associated with an unfavorable outcomes [1,2,4,17,18] but in our study only AKI and hypotension in low GCS contributed independently to increasing hospital mortality together with the TBI severity variables (low initial GCS, first worst CT and intracranial hypertension).

AKI has been considered an uncommon non-neurological complication of severe TBI and is not associated with increased mortality in these patients [3,4,19]. In our study, the incidence was 8%, similar to a recent study in which AKI was defined by the Risk, Injury, Failure, Loss, and End-stage Kidney (RIFLE) classification and associated with age and higher Acute Physiology and Chronic Health Evaluation (APACHE) III score [20]. The change in renal function from baseline is now considered a better measure of renal injury than single serum creatinine cutoff measurements [20,21]; we applied the latter in our study, which was conducted before the introduction of the RIFLE classification for acute kidney injury evaluation. The similarity in the incidence of AKI with the two assessment methods suggests that the inclusion of patients at an early stage of renal dysfunction did not account for the differences in identifying renal injury as a non-neurological risk factor for mortality in severe TBI. In our study, 13 of 17 patients with AKI (76%) died. In 10 of the 13 patients (77%) death occurred after day 7 of ICU admission. AKI may be related to hypotension during ICU, although in this study it was independently associated with hospital mortality. Improvements in neurological and non-neurological monitoring and management in the ICU during these years might contribute to decrease this complication. Restoring effective circulating volume, reducing renal injury from rhabdomyolysis and avoiding nephrotoxic drugs may prevent AKI initially [22], but larger series are needed to analyze risk factors and treatment of AKI in recent severe TBI patients, probably related to multiple organ failure.

Hypotension, both at admission and during the ICU stay, has been associated with poor prognosis in patients with severe TBI [17,18], so that we analyzed accurately. Duration of hypotension has also been considered one of the worst prognostic factors in these patients [1,18]. In our study, hypotension at some point during the ICU stay was frequent (44%). However, in contrast to other studies [3,5], it was not found to be an independent risk factor for mortality, although it increased mortality in patients with low GCS. Patients with low GCS (3 to 5) show global brain, brainstem or reticular system damage [23], which may involve the dysfunction of systemic homeostasis. This leads to non-neurological neurogenic dependent complications, which are difficult to distinguish because of the multiple conditions that these patients present, including neurogenic myocardial dysfunction and neurogenic pulmonary edema. The etiological hypothesis is that catecholamine release leads to these cardiopulmonary abnormalities. Dysregulated inflammatory mechanisms also appear to play a role in the development of the multiple organ dysfunction syndrome [5]. In our study, neurogenic causes appear to cause the hypotension that increases mortality in patients with low GCS. Despite using vasoactive drugs, a more aggressive resuscitation of patients who are at risk of hypotension, to improve outcome, requires further investigation. The bad neurological prognosis of these patients may be a limitation to using more aggressive life-sustaining therapies during their ICU stay.

Other complications, such as respiratory problems, were also frequently observed in patients with severe TBI in this study. Pneumonia was common (42%), as in other studies [3,4,24], but was not associated with increased mortality. Ventilator-associated pneumonia does not appear to raise in-hospital mortality in patients with severe TBI, but it prolongs mechanical ventilation, ICU and hospital length of stay, and increases the need for tracheostomy [25-27]. Severe respiratory failure occurred in 41% of patients, similar to another study [5]. Moderate and severe respiratory failure are poor prognostic factors [28-30], but in other studies they have not been associated with increased hospital mortality [19]. In the study by Zygun *et al. *neither SOFA-defined nor MOD-defined respiratory failure was significantly associated with increased hospital mortality [19]. In the study by Bratton *et al. *acute lung injury (ALI), PaO_2_/FiO_2 _< 300 was associated with the global severity of head injury (lower GCS scores) but not with specific anatomic lesions diagnosed by cranial CT scans [28]. In our study, severe respiratory failure was not independently associated with mortality, but it increased ICU stay. The measures proposed to prevent respiratory complications are: prevention of lung collapse and consolidation, prevention of lung infection, and acceleration of weaning from mechanical ventilation in the ICU [31]. The use of prone position, positive end-expiratory pressure (PEEP) and recruitment maneuvers have proved effective in improving tissue oxygenation with minimal negative effects of intracranial pressure and cerebral blood flow. Accurate monitoring of the physiologic cerebral function is warranted to minimize the possible negative effects on the brain induced by the maneuvers themselves [31-33]. Recently analyzed ventilator care bundles may be useful to prevent ventilator-associated pneumonia [34].

Intracranial hypertension is a poor prognostic factor in patients with severe TBI [1,18,35-39]. The presence of multiple trauma was not associated with mortality in this study. SOFA and MOD scales have been used to analyze non-neurological complications [5,6,19]. In one study, intracranial hypertension had higher maximum SOFA and delta SOFA, and there was a tendency for lower GCS to present higher maximum SOFA and delta SOFA which was not subsequently confirmed with the assessment of MOD [6]. However, the same authors found that the SOFA scoring system had superior discriminative ability and a stronger association with outcome than MOD with regard to hospital mortality and unfavorable neurological outcome [19]. In another study, patients with acute brain injury with GCS < 9 had a greater incidence of sepsis, respiratory failure and need for vasopressor therapy than those with GCS > 8 [40]. However, the relationship between non-neurological organ dysfunction and TBI severity has not always been demonstrated [7]. In our study, mortality depended mainly on the TBI initial severity (low GCS 3 to 5 and worst CT scan); as in previous studies, cardiovascular, respiratory and infectious problems were the most frequent non-neurological complications [3-5,19,40], but only AKI and hypotension in the low GCS group were independently associated with higher mortality.

This study has some limitations. This study was done in a single center. The definitions of non-neurological complications are not standardized; although a recent study tried to standardize data collection in TBI they did not define non-neurological complications [41]. The complications were associated with mortality, but each insult was not collected individually, so the particular impact of each one in secondary brain damage could not be evaluated. The episodes of hypotension were recorded from the first day of admittance in the ICU until the day of discharge, therefore the episodes could have appeared at any time during the period between admittance and discharge of the patients. Nor were the severity and timing of non-neurological complications recorded. However, it has been shown previously that time to development of these complications does not influence the mortality of patients with severe TBI [5], but the knowledge of the timing of these complications would be helpful to determine if these complications occurred in a pre-terminal phase or not. Withdrawal of life-sustaining therapies was not recorded.

## Conclusions

In summary, low initial GCS, first CT scan and intracranial hypertension are known neurological variables that determine mortality in severe TBI patients. AKI was the only non-neurological complication associated with ICU mortality. These findings add to previous studies that showed that non-neurological complications increase length of stay and morbidity in the ICU, but do not increase mortality, with the exception of AKI and hypotension in low GCS (3 to 5).

## Key messages

- GCS 3 to 5, first CT scan and intracranial hypertension are neurological variables that determine mortality in severe TBI patients.

- AKI and the association of low GCS with hypotension were the non-neurological complications independently associated with ICU mortality.

- Non-neurological complications increase length of stay and morbidity in ICU, but do not increase mortality.

## Abbreviations

AKI: acute kidney injury; ALI: acute lung injury; APACHE: Acute Physiology and Chronic Health Evaluation; ARDS: Acute Respiratory Distress Syndrome; AST: aspartate amino transferase; CI: confidence interval; CPP: cerebral perfusion pressure; CT: computed tomography; GCS: Glasgow Coma Scale; GOS: Glasgow Outcome Scale; ICP: intracranial pressure; MOD: multiple organ dysfunction; PEEP: positive end-expiratory pressure; RIFLE: Risk: Injury: Failure: Loss: and End-stage Kidney; SBP: systolic blood pressure; SIADH: Syndrome of Inappropriate Antidiuretic Hormone Secretion; SOFA: Sequential Organ Failure Assessment; SRF: severe respiratory failure; TBI: traumatic brain injury; TCDB: Traumatic Coma Data Bank.

## Competing interests

The authors declare that they have no competing interests.

## Authors' contributions

LC and JV conceived of the study and participated in its design. LC, JV and JH collected data. PM and CJ performed the statistical analysis. RM participated in the study coordination and helped to draft the manuscript. All authors read and approved the final manuscript.

## References

[B1] Andrews PJ Sleeman DH Statham PF McQuatt A Corruble V Jones PA Howells TP McMillan CS Predicting recovery in patients suffering from traumatic brain injury by using admission variables and physiological data: a comparison between decision tree analysis and logistic regression J Neurosurg 2002 16 326 336 10.3171/jns.2002.97.2.0326 12186460

[B2] Perel P Arango M Clayton T Edwards P Komolafe E Poccock S Roberts I Shakur H Steyerberg E Yutthakasemsunt S Predicting outcome after traumatic brain injury: practical prognostic models based on large cohort of international patients BMJ 2008 16 425 429 18270239 10.1136/bmj.39461.643438.25 PMC2249681

[B3] Piek J Chesnut RM Marshall LF van Berkum-Clark M Klauber MR Blunt BA Eisenberg HM Jane JA Marmarou A Foulkes MA Extracranial complications of severe head injury J Neurosurg 1992 16 901 907 10.3171/jns.1992.77.6.0901 1432133

[B4] Schirmer-Mikalsen K Vik A Gisvold SE Skandsen T Hynne H Klepstad P Severe head injury: control of physiological variables, organ failure and complications in the intensive care unit Acta Anaesthesiol Scand 2007 16 1194 1201 17711565 10.1111/j.1399-6576.2007.01372.x

[B5] Zygun DA Kortbeek JB Fick GH Laupland KB Doig CJ Non-neurologic organ dysfunction in severe traumatic brain injury Crit Care Med 2005 16 654 660 10.1097/01.CCM.0000155911.01844.54 15753760

[B6] Zygun DA Doig CJ Gupta AK Whiting G Nicholas C Shepherd E Conway-Smith C Menon DK Non-neurological organ dysfunction in neurocritical care J Crit Care 2003 16 238 244 10.1016/j.jcrc.2003.10.007 14691897

[B7] Zygun D Non-neurological organ dysfunction in neurocritical care: impact on outcome and etiological considerations Curr Opin Crit Care 2005 16 139 143 10.1097/01.ccx.0000155356.86241.c0 15758594

[B8] Lim HB Smith M Systemic complications after head injury: a clinical review Anaesthesia 2007 16 474 482 10.1111/j.1365-2044.2007.04998.x 17448059

[B9] Corral L Ventura JL Herrero JI Monfort JL Juncadella M Gabarros A Bartolome C Javierre C Garcia-Huete L Improvement in GOS and GOSE scores 6 and 12 months after severe traumatic brain injury Brain Inj 2007 16 1225 1231 10.1080/02699050701727460 18236198

[B10] Genarelli TA Spielman GM Langfitt TW Gildenberg PL Harrington T Jane JA Marshall LF Miller JD Pitts LH Influence of type of intracranial lesion on outcome from severe head injury J Neurosurg 1982 16 26 32 10.3171/jns.1982.56.1.0026 7054419

[B11] Maas AI Dearden M Teasdale GM Braakman R Cohadon F Iannotti F Karimi A Lapierre F Murray G Ohman J Persson L Servadei F Stocchetti N Unterberg A EBIC-guidelines for management of severe head injury in adults. European Brain Injury Consortium Acta Neurochir (Wien) 1997 16 286 294 10.1007/BF01808823 9202767

[B12] Maset AL Marmarou A Ward JD Choi S Lutz HA Brooks D Moulton RJ DeSalles A Muizelarr JP Turner H Pressure-volume index in head injury J Neurosurg 1987 16 832 840 10.3171/jns.1987.67.6.0832 3681422

[B13] Bernard GR Artigas A Brigham KL Carlet J Falke K Hudson L Lamy M LeGall JR Morris A Spragg R The American-European Consensus Conference on ARDS. Definitions, mechanisms, relevant outcomes, and clinical trial coordination Am J Respir Crit Care Med 1994 16 818 824 7509706 10.1164/ajrccm.149.3.7509706

[B14] Bone RC Balk RA Cerra FB Dellinger RP Fein AM Knaus WA Schein RM Sibbald WJ Definitions for sepsis and organ failure and guidelines for the use of innovative therapies in sepsis. The ACCP/SCCM Consensus Conference Committee. American College of Chest Physicians/Society of Critical Care Medicine Chest 1992 16 1644 1655 10.1378/chest.101.6.1644 1303622

[B15] Harrigan MR Cerebral salt wasting syndrome: a review Neurosurgery 1996 16 152 160 10.1097/00006123-199601000-00035 8747964

[B16] Corral L Herrero JI Monfort JL Ventura JL Javierre CF Juncadella M Garcia-Huete L Bartolome C Gabarros A First CT findings and improvement in GOS and GOSE scores 6 and 12 months after severe traumatic brain injury Brain Inj 2009 16 403 410 10.1080/02699050902788477 19301165

[B17] Chesnut RM Marshall SB Piek J Blunt BA Klauber MR Marshall LF Early and late systemic hypotension as a frequent and fundamental source of cerebral ischemia following severe brain injury in the Traumatic Coma Data Bank Acta Neurochir Suppl (Wien) 1993 16 121 125 8310858 10.1007/978-3-7091-9302-0_21

[B18] Marmarou A Anderson RL Ward JD Choi SC Young HF Eisenberg HM Foulkes MA Marshall LF Jane JA Impact of ICP instability and hypotension on outcome in patients with severe head trauma J Neurosurg 1991 16 S59 S66

[B19] Zygun D Berthiaume L Laupland K Kortbeek J Doig C SOFA is superior to MOD score for the determination of non-neurologic organ dysfunction in patients with severe traumatic brain injury: a cohort study Crit Care 2006 16 R115 10.1186/cc5007 16882348 PMC1750966

[B20] Moore EM Bellomo R Nichol A Harley N Macisaac C Cooper DJ The incidence of acute kidney injury in patients with traumatic brain injury Ren Fail 2010 16 1060 1065 10.3109/0886022X.2010.510234 20863210

[B21] Ricci Z Cruz D Ronco C The RIFLE criteria and mortality in acute kidney injury: a systematic review Kidney Int 2008 16 538 546 10.1038/sj.ki.5002743 18160961

[B22] Davenport A Management of acute kidney injury in neurotrauma Hemodial Int 2010 16 Suppl 1 S27 S31 21040416 10.1111/j.1542-4758.2010.00487.x

[B23] Laureys S Owen AM Schiff ND Brain function in coma, vegetative state, and related disorders Lancet Neurol 2004 16 537 546 10.1016/S1474-4422(04)00852-X 15324722

[B24] Bronchard R Albaladejo P Brezac G Geffroy A Seince PF Morris W Branger C Marty J Early onset pneumonia: risk factors and consequences in head trauma patients Anesthesiology 2004 16 234 239 10.1097/00000542-200402000-00009 14739794

[B25] Mascia L Zavala E Bosma K Pasero D Decaroli D Andrews P Isnardi D Davi A Arguis MJ Berardino M Ducati A Brain IT group High tidal volume is associated with the development of acute lung injury after severe brain injury: an international observational study Crit Care Med 2007 16 1815 1820 10.1097/01.CCM.0000275269.77467.DF 17568331

[B26] Zygun DA Zuege DJ Boiteau PJ Laupland KB Henderson EA Kortbeek JB Doig CJ Ventilator-associated pneumonia in severe traumatic brain injury Neurocrit Care 2006 16 108 114 10.1385/NCC:5:2:108 17099256

[B27] Rincon-Ferrari MD Flores-Cordero JM Leal-Noval SR Murillo-Cabezas F Cayuelas A Munoz-Sanchez MA Sanchez-Olmedo JI Impact of ventilator-associated pneumonia in patients with severe head injury J Trauma 2004 16 1234 1240 10.1097/01.TA.0000119200.70853.23 15625455

[B28] Bratton SL Davis RL Acute lung injury in isolated traumatic brain injury Neurosurgery 1997 16 707 712 10.1097/00006123-199704000-00009 9092843

[B29] Holland MC Mackersie RC Morabito D Campbell AR Kivett VA Patel R Erickson VR Pittet JF The development of acute lung injury is associated with worse neurologic outcome in patients with severe traumatic brain injury J Trauma 2003 16 106 111 10.1097/01.TA.0000071620.27375.BE 12855888

[B30] Kemp CD Johnson JC Riordan WP Cotton BA How we die: the impact of nonneurologic organ dysfunction after severe traumatic brain injury Am Surg 2008 16 866 872 18807680

[B31] Pelosi P Severgnini P Chiaranda M An integrated approach to prevent and treat respiratory failure in brain-injured patients Curr Opin Crit Care 2005 16 37 42 10.1097/00075198-200502000-00006 15659943

[B32] Koutsoukou A Perraki H Raftopoulou A Koulouris N Sotiropoulou C Kotanidou A Orfanos S Roussos C Respiratory mechanics in brain-damaged patients Intensive Care Med 2006 16 1947 1954 10.1007/s00134-006-0406-0 17053881

[B33] Mascia L Grasso S Fiore T Bruno F Berardino M Ducati A Cerebro-pulmonary interactions during the application of low levels of positive end-expiratory pressure Intensive Care Med 2005 16 373 379 10.1007/s00134-004-2491-2 15668765

[B34] Rello J Chastre J Cornaglia G Masterton R A European care bundle for management of ventilator-associated pneumonia J Crit Care 2011 16 3 10 10.1016/j.jcrc.2010.04.001 20537504

[B35] Fearnside MR Cook RJ McDougall P McNeil RJ The Westmead Head Injury Project outcome in severe head injury. A comparative analysis of pre-hospital, clinical and CT variables Br J Neurosurg 1993 16 267 279 10.3109/02688699309023809 8338647

[B36] Miller JD Ishii S, Nagai H, Brock M Significance and management of intracranial hypertension in head injury Intracranial Pressure 1983 Berlin: Springer-Verlag 44 55

[B37] Narayan RK Greenberg RP Miller JD Enas GG Choi SC Kishore PR Selhorst JB Lutz HA Becker DP Improved confidence of outcome prediction in severe head injury. A comparative analysis of the clinical examination, multimodality evoked potentials, CT scanning, and intracranial pressure J Neurosurg 1981 16 751 762 10.3171/jns.1981.54.6.0751 7241184

[B38] Stocchetti N Rossi S Buzzi F Mattioli C Paparella A Colombo A Intracranial hypertension in head injury: management and results Intensive Care Med 1999 16 371 376 10.1007/s001340050860 10342510

[B39] Vik A Nag T Fredriksli OA Skandsen T Moen KG Schirmer-Mikalsen K Manley GT Relationship of "dose" of intracranial hypertension to outcome in severe traumatic brain injury J Neurosurg 2008 16 678 684 10.3171/JNS/2008/109/10/0678 18826355

[B40] Mascia L Sakr Y Pasero D Payen D Reinhart K Vincent JL Extracranial complications in patients with acute brain injury: a post-hoc analysis of the SOAP study Intensive Care Med 2008 16 720 727 10.1007/s00134-007-0974-7 18175107

[B41] Maas AI Harrison-Felix CL Menon D Adelson D Balkin T Bullock R Engel DC Gordon W Langlois-Oman J Lew HI Robertson C Temkin N Valadka A Verfaellie M Wainwright M Wright DW Schwab K Standardizing Data Collection in Traumatic Brain Injury J Neurotrauma 2011 16 177 187 10.1089/neu.2010.1617 21162610 PMC3037806

